# PSMA PET Imaging in Glioblastoma: A Preclinical Evaluation and Theranostic Outlook

**DOI:** 10.3389/fonc.2021.774017

**Published:** 2021-11-17

**Authors:** Maximilian A. Kirchner, Adrien Holzgreve, Matthias Brendel, Michael Orth, Viktoria C. Ruf, Katja Steiger, Dennis Pötter, Lukas Gold, Marcus Unterrainer, Lena M. Mittlmeier, Enio Barci, Roland E. Kälin, Rainer Glass, Simon Lindner, Lena Kaiser, Jessica Maas, Louisa von Baumgarten, Harun Ilhan, Claus Belka, Johannes Notni, Peter Bartenstein, Kirsten Lauber, Nathalie L. Albert

**Affiliations:** ^1^ Department of Nuclear Medicine, University Hospital, Ludwig-Maximilians-Universität (LMU) Munich, Munich, Germany; ^2^ Department of Radiation Oncology, University Hospital, Ludwig-Maximilians-Universität (LMU) Munich, Munich, Germany; ^3^ Center for Neuropathology and Prion Research, Ludwig-Maximilians-Universität (LMU) Munich, Munich, Germany; ^4^ Institute of Pathology, Technische Universität München (TUM) School of Medicine, Technical University of Munich, Munich, Germany; ^5^ Department of Radiology, University Hospital, Ludwig-Maximilians-Universität (LMU) Munich, Munich, Germany; ^6^ Neurosurgical Research, Department of Neurosurgery, University Hospital, Ludwig-Maximilians-Universität (LMU) Munich, Munich, Germany; ^7^ German Cancer Consortium (DKTK), Partner Site Munich, German Cancer Research Center (DKFZ), Heidelberg, Germany; ^8^ Department of Neurosurgery, University Hospital, Ludwig-Maximilians-Universität (LMU) Munich, Munich, Germany

**Keywords:** Prostate specific membrane antigen (PSMA), ^18^F-PSMA-1007 PET, glioblastoma, GL261, preclinical, mouse

## Abstract

**Background:**

Prostate specific membrane antigen (PSMA) PET imaging has recently gained attention in glioblastoma (GBM) patients as a potential theranostic target for PSMA radioligand therapy. However, PSMA PET has not yet been established in a murine GBM model. Our goal was to investigate the potential of PSMA PET imaging in the syngeneic GL261 GBM model and to give an outlook regarding the potential of PMSA radioligand therapy in this model.

**Methods:**

We performed an ^18^F-PSMA-1007 PET study in the orthotopic GL261 model (n=14 GBM, n=7 sham-operated mice) with imaging at day 4, 8, 11, 15, 18 and 22 post implantation. Time-activity-curves (TAC) were extracted from dynamic PET scans (0-120 min p. i.) in a subset of mice (n=4 GBM, n=3 sham-operated mice) to identify the optimal time frame for image analysis, and standardized-uptake-values (SUV) as well as tumor-to-background ratios (TBR) using contralateral normal brain as background were calculated in all mice. Additionally, computed tomography (CT), *ex vivo* and *in vitro* ^18^F-PSMA-1007 autoradiographies (ARG) were performed.

**Results:**

TAC analysis of GBM mice revealed a plateau of TBR values after 40 min p. i. Therefore, a 30 min time frame between 40-70 min p. i. was chosen for PET quantification. At day 15 and later, GBM mice showed a discernible PSMA PET signal on the inoculation site, with highest TBR_mean_ in GBM mice at day 18 (7.3 ± 1.3 *vs*. 1.6 ± 0.3 in shams; *p*=0.024). *Ex vivo* ARG confirmed high tracer signal in GBM compared to healthy background (TBR_mean_ 26.9 ± 10.5 *vs*. 1.6 ± 0.7 in shams at day 18/22 post implantation; *p*=0.002). However, absolute uptake values in the GL261 tumor remained low (e.g., SUV_mean_ 0.21 ± 0.04 g/ml at day 18) resulting in low ratios compared to dose-relevant organs (e.g., mean tumor-to-kidney ratio 1.5E^-2^ ± 0.5E^-2^).

**Conclusions:**

Although ^18^F-PSMA-1007 PET imaging of GL261 tumor-bearing mice is feasible and resulted in high TBRs, absolute tumoral uptake values remained low and hint to limited applicability of the GL261 model for PSMA-directed therapy studies. Further investigations are warranted to identify suitable models for preclinical evaluation of PSMA-targeted theranostic approaches in GBM.

## 1 Introduction

Prostate specific membrane antigen (PSMA, synonyms: folate hydrolase I, glutamate carboxypeptidase II) targeted positron emission tomography (PET) imaging and radioligand therapy have gained increasing attention in recent years. Research efforts focusing on castrate resistant prostate cancer have resulted in the successful implementation of PSMA radioligand therapy into clinical routine, thus providing a valuable therapeutic option (1–6). PSMA overexpression can not only be found in prostate cancer but also in the neovasculature-associated endothelium of highly vascularized tumors such as renal cell carcinoma (7, 8) and glioblastoma (9–12). Glioblastoma (*Glioblastoma multiforme*, GBM) is the most frequent malignant primary brain tumor and highly aggressive with median survival being less than 20 months under standard therapy consisting of surgery, radiotherapy and chemotherapy with temozolomide (13–16). PSMA overexpression in GBM provides new options for PSMA based imaging and especially therapy that are desperately needed in clinical management.

Few studies with small numbers of patients have shown that PSMA PET imaging of glioma and glioblastoma is promising, showing high contrast of tumor and healthy brain tissue (17–21). However, there is little data available on PSMA radioligand therapy in GBM. Especially in GBM mouse models, data both for PSMA PET imaging and for PSMA radioligand therapy are lacking despite their potential in contributing to the understanding of PSMA pathophysiology in GBM. Hence, we investigated PSMA PET imaging in a murine GBM model in order to more thoroughly understand PSMA PET tracer uptake of GBM and to evaluate opportunities for potential PSMA radioligand therapy approaches in this model.

Here, we performed dynamic and static ^18^F-PSMA-1007 PET scans on GBM-bearing mice (murine GL261 cell line) and sham operated mice amounting to a total of 69 PET scans. In addition, *in vitro* uptake assays were conducted including GL261 cells, human U87 GBM cells (as the most common human GBM cell line) and LNCaP and PC-3 cells as PSMA positive and negative controls, respectively. Autoradiography (ARG) and computed tomography (CT) were used to confirm PSMA signal and tumor location. Uptake specificity was assessed with a competitive binding assay.

## 2 Material And Methods

### 2.1 Study Design

In this multimodal preclinical study, *in vivo* and *in vitro* experiments including longitudinal ^18^F-PSMA-1007 PET imaging were performed to evaluate PSMA as a potential theranostic target in the most common murine syngeneic GBM model GL261. All animal experiments were performed in accordance to the FELASA guidelines and the German Animal Welfare Act and were reviewed and approved by the local regulatory authority, *Regierung von Oberbayern*.

In a first cohort, 4 tumor bearing mice (GBM mice, GL261 cell line) and 3 sham mice received dynamic ^18^F-PSMA-1007 PET imaging (0-120 min p. i.; 13.13 ± 1.44 MBq ^18^F-PSMA-1007 per mouse) at day 15 or 18 post implantation to evaluate ^18^F-PSMA-1007 uptake kinetics including time-activity-curves (TAC) and to determine an optimal time frame for following static PET imaging.

A second cohort with a total of 12 mice (8 GBM, 4 sham mice) received longitudinal static ^18^F-PSMA-1007 PET imaging (40-70 min p. i.; 13.32 ± 2.54 MBq ^18^F-PSMA-1007) at day 4, 8, 11, 15, 18 and two mice also at day 22 after tumor implantation. On day 4 the measurements of only 5 GBM and 3 sham mice were included in statistical analyses due to incomplete data acquisition or tracer injection of 4 mice (3 GBM, 1 sham) (also see **Table 1**). For the same reasons 1 GBM mouse on day 18 had to be excluded from statistical analysis. 3 mice had to be euthanized before scheduled due to critical health condition on day 16, 18 and 21.

**Table 1:** Overview study design.

Day	PSMA PET		CT		PSMA ARG	
	n	(GBM | sham)	n	(GBM | sham)	n	(GBM | sham)
**Dynamic imaging**						
**15^a^**	3	(2 | 1)	7	(5 | 2)	2	(2 | 0)
**18**	4	(2 | 2)	4^b^	(2 | 2)	2	(2 | 0)
	**7**	**(4 | 3)**	**11**	**(7| 4)**	**4**	**(4 | 0)**
**Longitudinal imaging**						
**4^c^**	8	(5 | 3)	12	(8 | 4)	−	
**8**	12	(8 | 4)	12	(8 | 4)	−	
**11**	12	(8 | 4)	12	(8 | 4)	−	
**15**	12	(8 | 4)	12	(8 | 4)	1	(0 | 1)
**18^c^**	10	(7 | 3)	11^d^	(8 | 3)	8	(6 | 2)
**22**	2	(1 | 1)	2	(1 | 1)	2	(1 | 1)
	**56**	**(37 | 19)**	**61**	**(41 | 20)**	**11**	**(7 | 4)**
**Competitive binding**						
**16**	2	(2 | 0)	−		2	(2 | 0)
**Total**	**65**	**(43 | 22)**	**72**	**(48 | 24)**	**17**	**(13 | 4)**

^a^Due to technical difficulties only 3 out of 7 PET scans could be acquired successfully. CTs were performed as planned. ^b^CTs were performed the day prior to PET scans. ^c^From the total of 12 mice (8 GBM, 4 sham) in the longitudinal imaging cohort the measurements of 4 mice (3 GBM, 1 sham) on day 4 and 1 GBM mouse on day 18 were excluded from statistical analysis due to incomplete data acquisition or tracer injection ^d^One mouse had to be euthanized after CT and received no PET.

All mice received contrast-enhanced CT between 2-4 hours prior to each PET. Mice were sacrificed immediately after their last PET scan to receive *ex vivo* and *in vitro* ^18^F-PSMA-1007 ARG as well as histopathologic staining. **Table 1** gives a summary of the study design.

Two additional mice were used for *in vivo* competitive binding assays to evaluate ^18^F-PSMA-1007 uptake specificity as described below.

### 2.2 Cell Preparation

The GL261 murine glioblastoma cell line was obtained from the National Cancer Institute (NCI, Frederick, MD, USA) and cultured at 37°C and 7.5% CO_2_ in DMEM supplemented with 10% heat-inactivated fetal calf serum (FCS), 100 units/ml penicillin, and 0.1 mg/ml streptomycin (all from Thermo Scientific, Schwerte, Germany). The U87 human glioblastoma cell line was purchased from Cell Line Service (CLS, Heidelberg, Germany) and cultured under identical conditions. The human prostate cancer cell lines PC-3 and LNCaP were also purchased from CLS and were cultured in DMEM/F12 GlutaMAX medium supplemented with 5% FCS, 100 units/ml penicillin, and 0.1 mg/ml streptomycin at 37°C and 7.5% CO_2_, or in RPMI 1640 medium supplemented with 10% FCS, 100 units/ml penicillin, 0.1 mg/ml streptomycin, and 1% HEPES (all from Thermo Scientific) at 37°C and 5% CO_2_, respectively. The identity of all human cell lines was confirmed by short tandem repeat (STR) typing (service provided by the DSMZ, Braunschweig, Germany). All cell lines were passaged in 2-3 d intervals at 1:10 ratio using Trypsin/EDTA (Thermo Scientific).

### 2.3 *In Vitro* Evaluation

To evaluate ^18^F-PSMA-1007 internalization and binding (in the following referred to as *uptake*) in the murine glioblastoma cell line GL261 and human glioblastoma cell line U87, an *in vitro* uptake assay was conducted. PSMA positive LNCaP and PSMA negative PC-3 human prostate cancer cell lines served as positive and negative controls, respectively.

Three different cell densities (6 × 10^5^, 8 × 10^5^, and 1 × 10^6^ cells per well) of GL261, U87, LNCaP and PC-3 cells were seeded 24 h before the experiment in 6-well plates. The cells in each well were then incubated with 200 µl of ^18^F-PSMA-1007 plus 600 µl of the corresponding cell medium (corresponding to 89.40 kBq of ^18^F-PSMA-1007 per well) for 60 or 120 min at 37°C and 5% CO_2_. After incubation, supernatant was disposed and cells were washed with 800 µl phosphate-buffered saline (PBS). To measure the ^18^F-PSMA-1007 uptake, cells were subsequently incubated twice for 10 min with 800 µl of 1M NaOH and supernatant was collected in measurement tubes. Radioactivity was measured using a gammacounter and given in counts per minute (CPM). As a reference for PSMA uptake, six tubes with 200 µl ^18^F-PSMA-1007 plus 600 µl PBS were used to estimate initially added tracer activity and correct for pipetting errors. Uptake was calculated as a fraction of initially added tracer activity (CPM/CPM_IA_, [%]). The assay was conducted in duplicates.

### 2.4 *In Vivo* Evaluation

#### 2.4.1 Animal Model and Tumor Implantation

Female C57BL/6 mice (8-10 weeks old) were obtained from Charles River (Sulzfeld, Germany) and housed in ventilated cages in a pathogen-free animal facility with a 12 h day/night cycle. Mice were provided with standard rodent food and water *ad libitum*, and inspected on a daily basis.

Implantation was performed at day 0. All other times given refer to the day after implantation. For orthotopic implantation GL261 cells were cultured as described above and detached by trypsinization, washed twice in phosphate-buffered saline (PBS, Thermo Scientific), counted by using a Neubauer counting chamber, and resuspended at a final concentration of 10,000 cells/μl. 1 μl (10,000 cells) was used for inoculation. Implantation was performed as previously described (22). In brief, after pre-medication and anesthesia (200 µg/g metamizol, 100 µg/g ketamine and 10 µg/g xylazine, all WDT, Garbsen, Germany) heads were mounted on a stereotactic frame (David Kopf Instruments, Tujanga, CA, USA). Skulls were exposed by a longitudinal skin incision, and a hole was drilled 1.5 mm laterally and 1 mm anterior to the *bregma*. After stereotactical injection of 10,000 GL261 cells (GBM mice) or 1 µl saline for control (sham mice) into the right *striatum*, the skin was sutured, and mice were monitored until regaining consciousness.

#### 2.4.2 Positron Emission Tomography (PET)

Tracer injection and PET imaging (Siemens Inveon DPET, Siemens, Erlangen, Germany) were performed on a heating plate and under anesthesia (1.5% isoflurane at 3.5 L/min in oxygen). Mice on average received a bolus injection of 13.30 MBq ± 2.44 ^18^F-PSMA-1007 in 200 µl of saline into the tail vein. ^18^F-PSMA-1007 was synthesized as previously described (23, 24). Transmission for scatter and attenuation correction was obtained using a rotating ^57^Co point source. Image reconstruction was done as described previously (25): The procedure contains three dimensional ordered subset expectation using four iterations and 12 subsets followed by a maximum a posteriori algorithm with a beta value of 0.01 and 32 iterations. Images were corrected for scatter and attenuation and revised for ^18^F decay. Voxel dimension was 0.40 mm × 0.40 mm × 0.79 mm using a zoom factor of 1.0 and a 256 × 256 × 161 matrix.

Dynamic PET imaging was performed with emission recorded 0-120 min p. i. followed by a 15 min transmission scan and reconstructed with 31 frames. Static PET images were acquired 40-70 min p. i. with 15 min transmission recorded prior or after emission with one 30 min frame reconstructed.

#### 2.4.3 Contrast-Enhanced Computed Tomography (CT)

For the first cohort of mice (dynamic PET) contrast-enhanced conebeam computed tomography scans were performed as previously described (22) using a small animal radiation research platform (SARRP, X-strahl, Camberley, Great Britain). Briefly, mice received an intravenous 300 µl imeron-300 (equivalent to 90 mg iodine, Bracco, Konstanz, Germany) bolus injection 3 min prior to CT acquisition for contrast enhancement.

For the second cohort of mice (static PET) the Molecubes X-CUBE (Molecubes, Belgium) was used. Contrast agent injection and anesthesia were performed as described above.

#### 2.4.4 Image Analysis

Image analysis was done using PMOD (PMOD Technologies Limited, Switzerland). Reconstructed PET images were fused manually onto their respective CT.

For volume-of-interest (VOI) based PET image analysis tumor and background VOIs were defined. The background was defined as a fixed volume (30 mm^3^) background (BG) VOI in the unaffected tumor free contralateral hemisphere for all mice. For all GBM mice with PSMA signal above BG at the site of implantation, an individual tumor VOI using a 50% isocontour threshold (Iso_50_) was generated. For sham mice and GBM mice with no PSMA signal above BG at the site of implantation, a general tumor (GT) VOI with a fixed volume of 35 mm^3^ was defined and set in the right hemisphere. For parotid glands, bone marrow and kidneys VOIs were generated similarly using a 50% isocontour threshold.

Maximum and mean standardized uptake values (SUV_max_, SUV_mean_) were determined normalized by body mass and applied tracer activity, given in g/ml. Tumor-to-background ratios (TBR) were calculated by dividing SUV_mean_ and SUV_max_ of the tumor VOIs by the SUV_mean_ of the BG VOI (TBR_mean_ and TBR_max_, respectively). Tumor-to-kidney (TKR), -parotid gland (TPR) and -bone marrow (TBmR) ratios were calculated accordingly and determined for day 15 after implantation in the longitudinal cohort. SUVs and TBRs in dynamic images were assessed using time weighted summation images 40-70 min p. i. Time-activity-curves for 0-120 min p. i. were extracted and used for kinetic analysis.

#### 2.4.5 Autoradiography (ARG)

For *ex vivo* ARG mouse brains were removed from the skull and placed into a base mold embedded in cryo-matrix immediately after euthanization (< 5 min after end of PET scan). After snap freezing the brains for 5 min in a -80°C refrigerator, they were fixed in a Leica CM 1510-1 Cryostat (Leica Microsystems, Nussloch, Germany) at -20°C. Mouse brains were cut horizontally in 16 µm cryosections and every 24^th^, 25^th^ and 26^th^ section were mounted onto a glass slide. The slides were covered for a minimum of 12 hours with an imaging plate (Fujifilm; BAS cassette2 2025).

For *in vitro* ARG the tissue was covered with a solution of ^18^F-PSMA-1007 and TRIS-buffer solution (approx. 60 kBq/µl). After incubation for 1 h, the slides were washed in TRIS-buffer again, dried and covered with an imaging plate.

The plates were scanned at 25 µm resolution using Raytest equipment (CR 35 Bio, Dürr Medical, Germany). Image analysis was done using AIDA image analyzing software (V450). Regions-of-interest (ROI) were determined using the isocontour tool. A background ROI was defined manually in the contralateral tumor free hemisphere and TBR_mean_ was calculated.

#### 2.4.6 Histopathology

16 µm cryosections were prepared as described above and mounted onto a glass slide. Haematoxylin and eosin (H&E) staining was performed according to a standard protocol. An additionally performed CD31 immunohistochemical staining is reported in the **Supplementary Material**.

#### 2.4.7 Competitive Binding Assay

5 mg cold PSMA-1007 reference standard (ABX, Radeberg, Germany) were dissolved in 48.5 µl dimethyl sulfoxide (DMSO) and 4.8 ml water. 2 GBM mice received 100 µl of the solution (1 mMol, 1% DMSO, corresponding to 100 nmol PSMA-1007 reference standard) through the tail vein immediately prior (< 20 s) to injection with hot ^18^F-PSMA-1007 (20.06 ± 0.38 MBq, molar activity 106.62 MBq/nmol). Static PET scans were performed on day 16. Mice were euthanized immediately after the PET scan for *ex vivo* autoradiography. Tumor signal and kidney signal in PET scans were compared to the unblocked GBM mice of the dynamic imaging cohort (n=4). To test whether the blocking was successful kidney uptake was assessed and compared.

#### 2.4.8 Statistical Analysis

Statistical analysis was done using SPSS (IBM, version 21.0). Differences in SUV between GBM and sham mice were compared using Student’s t-tests. For differences in TBR values Mann-Whitney-U-tests were used. A *p*-value of less than 0.05 was considered significant.

## 3 Results

### 3.1 *In Vitro* Studies

**Figure 1:**
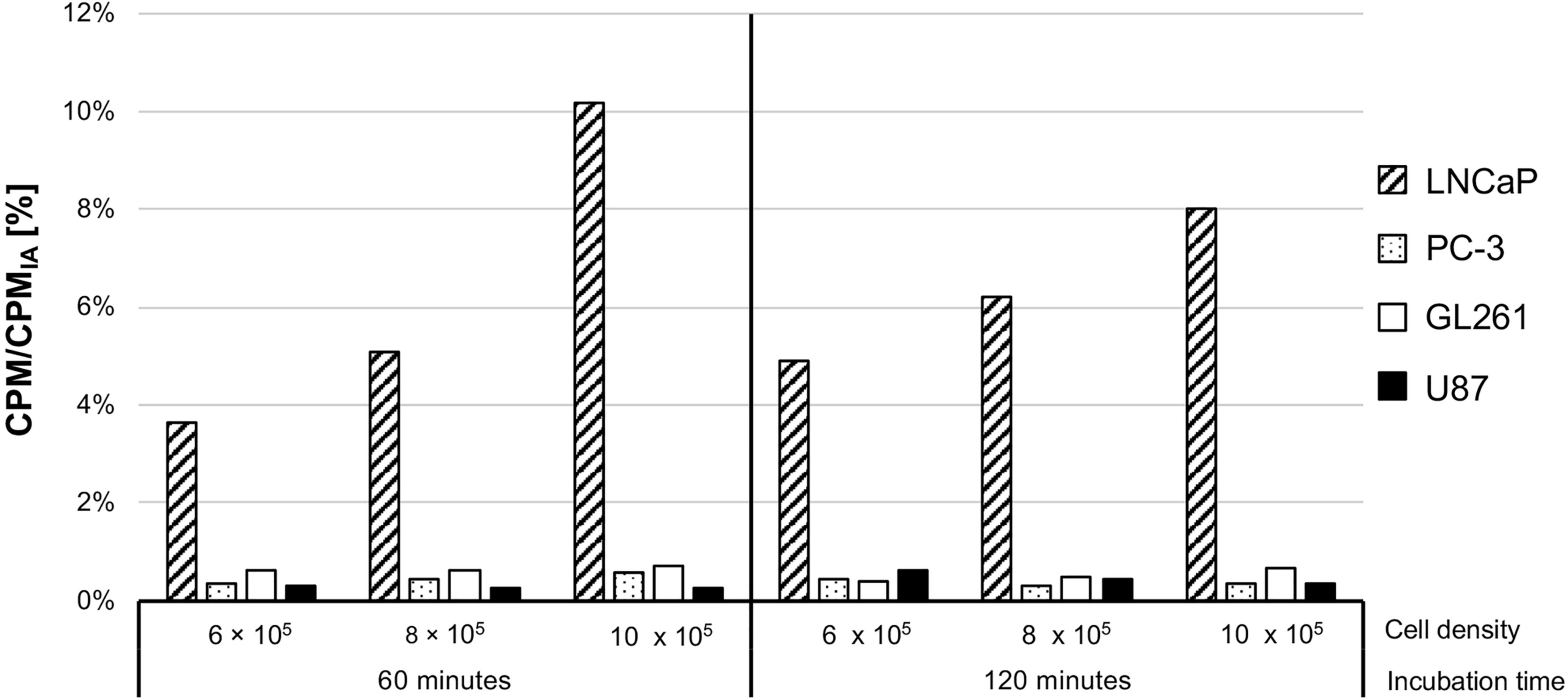
*In vitro* ^18^F-PSMA-1007 uptake. CPM/CPM_IA_: Internalization and binding given as a fraction of initially added activity. LNCaP and PC-3 cells served as PSMA positive and negative controls, respectively.

Uptake of ^18^F-PSMA-1007 is given as a fraction of initially added activity (CPM/CPM_IA_). **Figure 1** shows the uptake after 60 and 120 min tracer incubation for different cell densities respectively.

LNCaP cells showed the highest ^18^F-PSMA-1007 uptake independent of cell densities and incubation times ranging from 3.66% (60 min, 6 × 10^5^ cells per well) to 10.17% (60 min, 1 x 10^6^ cells per well). U87 and GL261 glioblastoma cell lines showed similar ^18^F-PSMA-1007 uptake to PSMA negative PC-3 cells for all densities and incubation times, with maximum uptake of 0.59% for PC-3 (60 min,1 x 10^6^ cells per well), 0.64% for U87 (120 min, 6 × 10^5^ cells per well) and 0.73% for GL261 (60 min, 1 x 10^6^ cells per well).

### 3.1 *In Vivo* Studies

#### 3.1.1 Dynamic PET Scans

**Figure 2:**
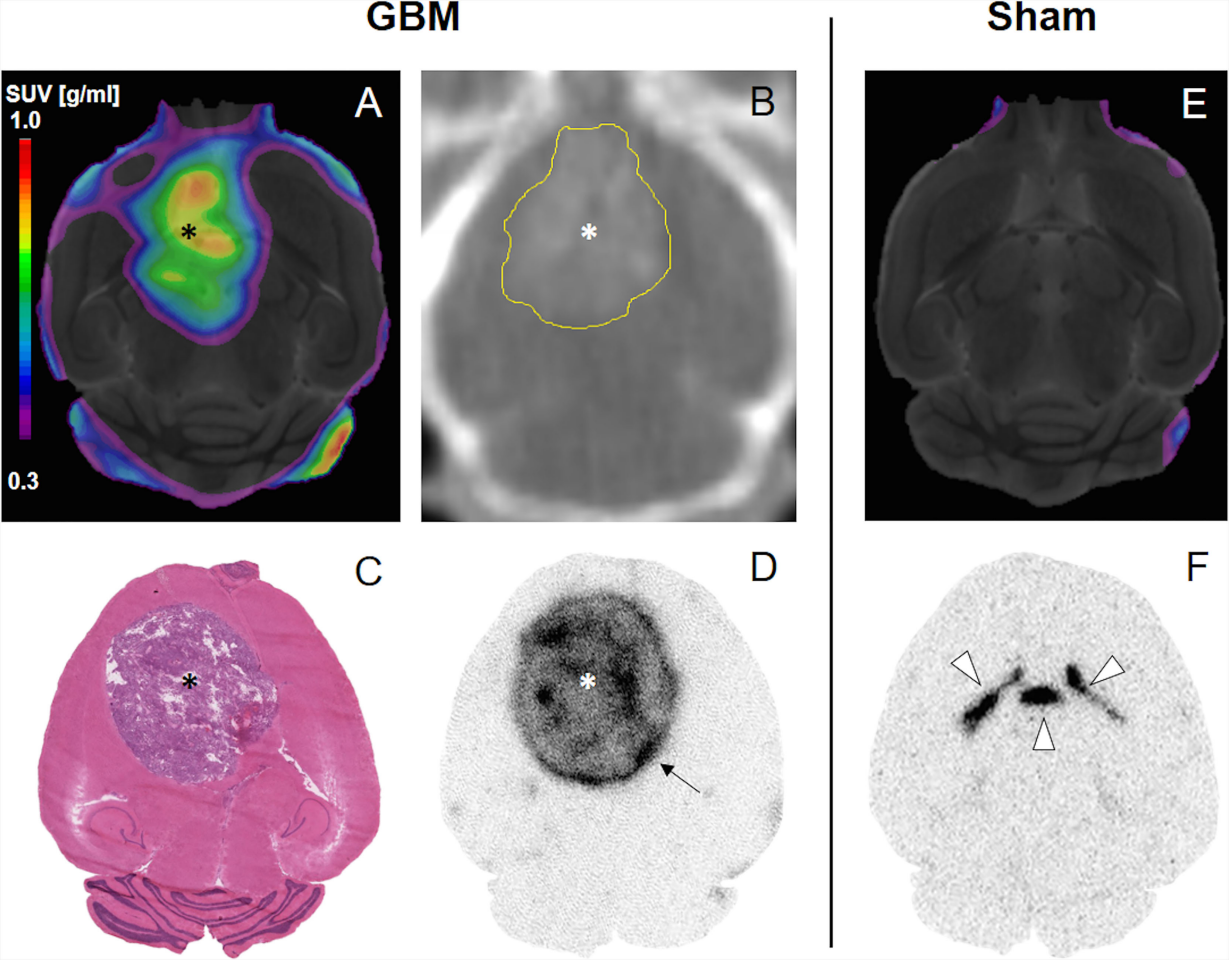
^18^F-PSMA-1007 PET **(A, E)**, CT **(B)**, H&E **(C)** and *ex vivo* ARG **(D, F)**. PET images were fused onto an MRI template. Asterisk, tumor. Yellow delineation in **(B)** for better visibility. Arrow, Increased peripheral signal. Arrowheads, ventricles; circumventricular organs.

All GBM mice of the dynamic imaging cohort (n=4) showed an increased PSMA PET signal at the site of implantation (**Figure 2A**); average SUV_max_ and SUV_mean_ for the entire group using the Iso_50_ tumor VOI were 0.67 ± 0.02 g/ml and 0.37 ± 0.08 g/ml, respectively. Healthy brain tissue on the other hand showed very low PSMA signal in PET images resulting in positive tumor contrast (e.g., TBR_mean_ 6.9 ± 1.5, 40-70 min p. i., see time-activity-analysis below for selection of time frame).

Sham mice showed no increased signal at the site of implantation compared to healthy brain tissue (*p*=0.858, see **Figure 2E**); the signal in sham mice was lower compared to GBM mice with TBR_mean_ 1.0 ± 0.1 (*p*=0.057), SUV_max_ 0.25 ± 0.06 g/ml (*p*=0.005), and SUV_mean_ 0.06 ± 0.01 g/ml (*p*=0.002).

Contrast enhanced CT (**Figure 2B**) and H&E staining (**Figure 2C**) confirmed the existence and location of the tumor.

#### 3.1.2 Analysis of Time-Activity-Curves

**Figure 3:**
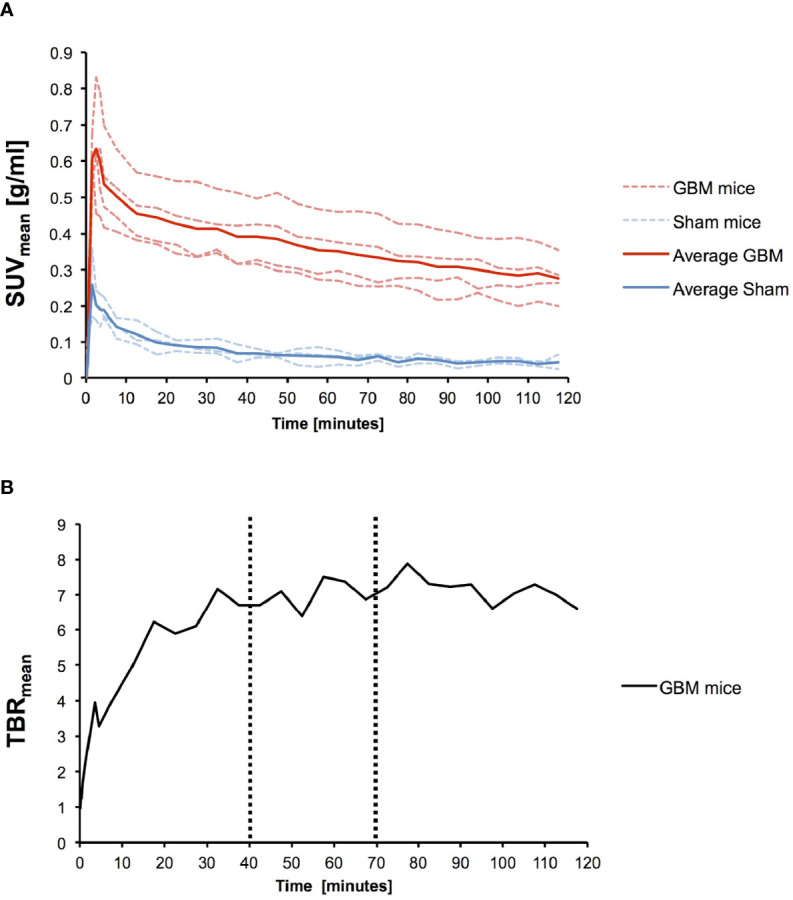
Time-activity-curves (TAC). **(A)** Individual TACs for n = 4 GBM (red dotted line) mice and n = 3 sham mice (blue dotted line) as well as the average SUVmean for GBM (red solid line) and sham mice (blue solid line) are given. **(B)** TBR_mean_ increased and stabilized after 40 min (max. 7.9 at 77.5 min). Therefore a 40-70 min time frame was chosen for further static scans.

GBM and sham mice showed similar tracer kinetics within the site of implantation (see TACs, **Figure 3A**). The PSMA signal in the Iso_50_ VOIs of GBM mice showed an early peak with time-to-peak (TTP) between 1.5 and 3.5 min followed by a decreasing kinetic. At the respective peak times, SUV_max_ ranged from 1.49 to 1.98 g/ml (average SUV_max_ at TTP: 1.69 ± 0.19 g/ml) and SUV_mean_ ranged from 0.61 to 0.83 g/ml (average SUV_mean_ at TTP: 0.54 ± 0.09 g/ml).

All three sham mice showed TTP of 1.5 min. At TTP, SUV_max_ ranged from 0.78 to 1.34 g/ml (average SUV_max_ at TTP: 1.00 ± 0.24 g/ml) and SUV_mean_ ranged from 0.17 to 0.36 g/ml (average SUV_mean_ at TTP: 0.26 ± 0.08 g/ml).

Bone marrow and parotid gland in GBM mice also showed early TTP but with average peak SUV_mean_ being considerably higher with 1.89 ± 0.98 g/ml for bone marrow and 1.22 ± 0.44 g/ml for parotid gland. Average kidney uptake in GBM mice increased over time and showed SUV_mean_ > 14.00 g/ml 1 h p. i. (see **Supplementary Material**).

TBR_mean_ for GBM mice increased over time with a maximum value of 7.9 at 77.5 min p. i. (**Figure 3B**). TBRs reached a plateau beginning approximately 40 min p. i. Therefore the authors chose a time frame of 40 – 70 min p. i. for VOI-based uptake quantification and further static scans. A 30 min time frame was chosen to correct for possible volatility in image acquisition.

#### 3.1.3 Longitudinal Imaging

**Figure 4:**
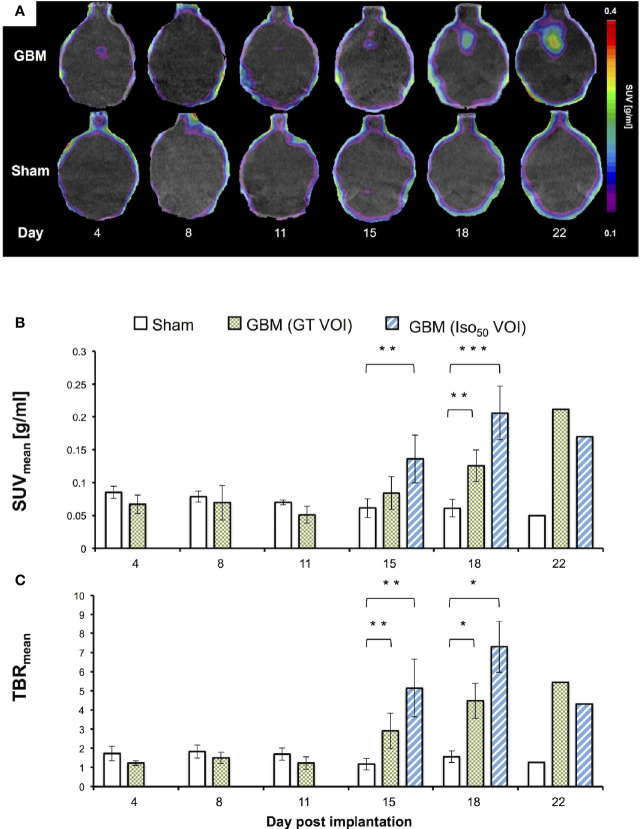
Longitudinal ^18^F-PSMA-1007 PET image fused on respective CT **(A)**, SUV_mean_ **(B)** and TBR_mean_ **(C)**. GT VOI, General tumor VOI. Day 4: (n = 5 GBM mice, n = 3 sham mice); day 8, 11 and 15: (all 8 GBM | 4 sham); day 18: (6 GBM | 3 sham); day 22 (1 GBM | 1 sham). *p < 0.05. **p < 0.01. ***p < 0.001.

**Figure 4A** gives example PET scans of one GBM and one sham mouse from day 4 to 22. **Figure 4B** gives the SUV_mean_ for GBM and sham mice at the respective PET scan date.

All longitudinally scanned GBM mice showed an increase in PSMA signal over time at the site of implantation while sham mice showed no increase (**Figure 4B**). PET signal increased at day 15 and later with visually identifiable tumors for all GBM mice (n=8). The individual Iso_50_ VOI was used in addition to the GT VOI to determine SUV_mean_ for day 15 and 18. From day 15 to 18, SUV_mean_ rose from 0.08 ± 0.02 g/ml to 0.13 ± 0.02 g/ml for the GT VOI and from 0.14 ± 0.04 g/ml to 0.21 ± 0.04 g/ml for the Iso_50_ VOI respectively. At day 15 SUV_mean_ for the Iso_50_ VOI differed significantly from the signal in the implantation location in sham mice (*p*=0.001). At day 18 SUV_mean_ showed a significant difference for GT VOI (*p*=0.003) and the Iso_50_ VOI (*p*<0.001).

Accordingly, GBM mice TBR_mean_ were constant at day 4, 8, and 11 and increased at day 15 and 18 (**Figure 4C**). TBR_mean_ for day 15 and 18 were 2.9 ± 0.9 and 4.5 ± 0.9 for the GT VOI and 4.8 ± 1.5 and 7.3 ± 1.3 for the Iso_50_ VOI respectively. The TBR_mean_ in GBM mice were significantly higher than the TBR_mean_ in sham mice at day 15 (*p*=0.004 for both VOIs) and 18 (*p*=0.024 for both VOIs).

Average Tumor-to-kidney, -bone marrow and -parotid gland were determined in GBM mice for day 18. TKR_mean_ was 1.5 × 10^-2^ ± 0.5 × 10^-2^, TBmR_mean_ 0.4 ± 0.1 and TPR_mean_ 0.1 ± 0.1.

#### 3.1.4 Autoradiography

**Figure 5:**
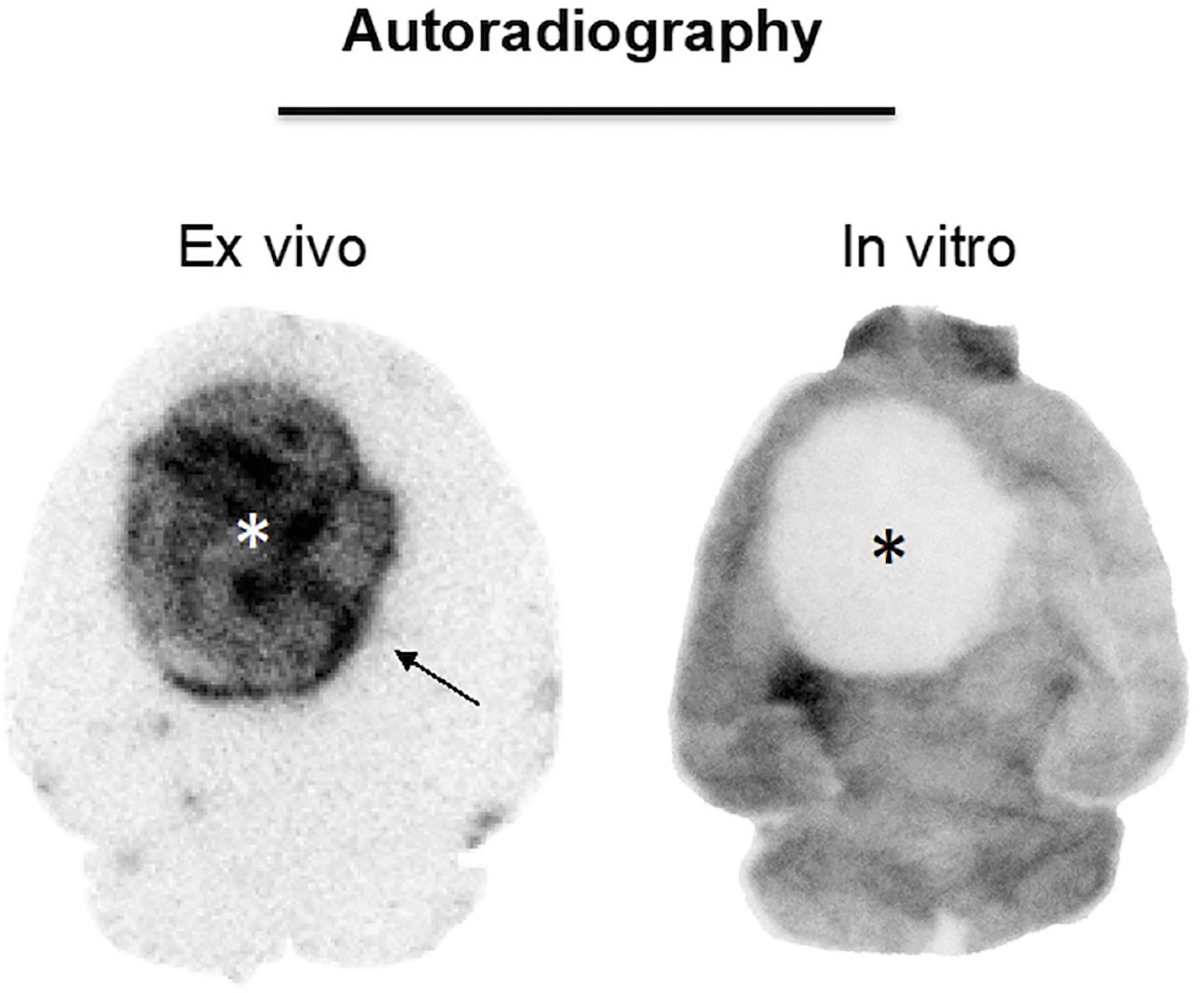
^18^F-PSMA-1007 *ex vivo* ARG and *in vitro* ARG in GBM. Asterisk, tumor. The *ex vivo* ARG (left) gave higher signal in tumor tissue than in the healthy brain tissue. The *in vitro* ARG on the other hand showed lower signal in the tumor tissue than in the healthy brain tissue.

In concordance with the impressions provided by PET scans, tumors in *ex vivo* ARG showed a strong signal whereas healthy brain tissue barely showed any uptake (**Figures 2D**, **5**). Interestingly, an increased signal at the periphery of the tumors could be found in 6 of 10 GBM mice (arrows in **Figure 2D** and **Figure 5**). Sham mice displayed an increased ventricular uptake and showed no signal in the site of implantation (**Figure 2F**). TBR for GBM and sham mice differed significantly with 26.9 ± 10.5 and 1.6 ± 0.7 respectively (*p*=0.002).

In *in vitro* ARG, by contrast, the tumor uptake was lower than the uptake in healthy brain tissue (**Figure 5**). It appeared that there was even no uptake at all in the respective lesion.

#### 3.1.5 Competitive Binding Assay

Surprisingly, the signal at the site of implantation in blocked mice (average SUV_mean_ 0.71 ± 0.24 g/ml) was higher than in mice that received no blocking (average SUV_mean_ 0.38 ± 0.08 g/ml). Kidney uptake on the other hand was lower in blocked mice as expected.

## 4 Discussion

Our results show that PSMA PET imaging is feasible in a GBM mouse model. We were able to confirm the high tumor-to-background-ratios that could be shown in human patients. Standardized-uptake-values on the other hand were low and hint to a limited eligibility of the GL261 model for an application of PSMA radioligand therapy.

As previous data has shown, PSMA expression in GBM is most likely not primarily related to tumor cells like in prostate cancer, but mainly occurs in the tumor-associated neovasculature (9, 26, 27). In concordance with those findings, we were able to show that one of the most common murine GBM cell lines, GL261, and one of the most common human GBM cell lines, U87, (i. e. both non-endothelial tumor cells) do not show any *in vitro* ^18^F-PSMA-1007 uptake (**Figure 1**). It has to be noted, that the uptake in our *in vitro* experimental setting was not corrected for unspecific uptake and might also measure tracer that is bound to the cellular surface. Nonetheless, the measured activity in GL261 and U87 cells was similar to the uptake of the negative control and can be interpreted to show no uptake. Liu et al. (2011) (28) and Nguyen et al. (2016) (29) were able to show that tumor cell medium could induce PSMA expression *in vitro* in human endothelial umbilical vein cells, which might be a promising step in establishing *in vitro* models for the study of PSMA uptake in GBM. However, up to now, only *in vivo* models provide a sufficient blueprint of the tumor neovasculature and its complex interplay with the tumor microenvironment.

In the *in vivo* study evaluating ^18^F-PSMA-1007 PET scans in one of the most commonly used syngeneic GBM mouse model (using the GL261 cell line), visual detection of GBM was clearly feasible (**Figure 2**) resulting in TBRs up to 7.3. Additionally, in the longitudinal PET imaging cohort the ^18^F-PSMA-1007 signal in GBM mice steadily increased over time, both in intensity and extent (e.g., see **Figure 4**). These results are in concordance with findings in glioblastoma patients that also show high TBR values in PSMA PET (30, 31). The high TBRs in PET were confirmed by high tumor-to-background contrast in *ex vivo* ARG. Interestingly, high resolution *ex vivo* ARG showed a slightly increased uptake in the tumor periphery, which has been previously described in a GBM model in rats by Oliveira et al. (2020) (32). The authors relate this enhanced uptake in the tumor periphery to astrocytic activation, which has yet to be confirmed for the GL261 model.

Notably, the level of TBR values in the current ^18^F-PSMA-1007 PET study was higher than the average TBR values obtained on *O*-(2-^18^F-fluoroethyl)-L-tyrosine (^18^F-FET) PET imaging, the current gold standard for glioma PET imaging (33) (e. g., TBR_max_ on ^18^F-FET PET in the GL261 GBM model = 3.2 (25), *vs*. 11.3 on ^18^F-PSMA-1007 PET). Likewise, in human patients ^18^F-FET PET imaging results in lower TBRs than reported for PSMA PET imaging (34). Although ^18^F-PSMA-1007 PET imaging of GBM was associated with high TBRs using the GL261 model, the absolute uptake values were comparably low (e. g., SUV_mean_ 0.21 ± 0.04 g/ml at day 18). Instead, the PSMA uptake in bone marrow, parotid gland and kidney was considerably higher than in the tumor, resulting in very low tumor-to-kidney, tumor-to-bone marrow and tumor-to-parotid gland ratios (TKR_mean_ 1.5 × 10^-2^ ± 0.5 × 10^-2^, TBmR_mean_ 0.4 ± 0.1 and TPR_mean_ 0.1 ± 0.1; see **Supplementary Material**).

Time-activity-curves of dynamic ^18^F-PSMA-1007 PET showed no difference between GBM and sham mice in the kinetic uptake pattern (**Figure 3**). The early TTP and subsequent decreasing kinetics in both GBM and sham mice indicate a continuous clearance of ^18^F-PSMA-1007. In comparison, TACs of prostate cancer tumor models show increasing kinetics and retention of PSMA (23, 35). Similarly, a recent case report (36) showed an increasing uptake of ^177^Lu-PSMA-617 within 24 hours in the case of a glioblastoma patient with a slow clearance up to 14 days after injection. Tumor-to-organ ratios in LNCaP tumor bearing mice on the other hand have been reported to be much higher (e.g. tumor-to-bone marrow > 20) with accumulation of tracer in the tumor tissue over time (37). Although our data does not include ^18^F-PSMA-1007 PET acquisitions later than 120 min p. i., the unfavorable tracer kinetics in combination with an overall low SUV advocate against the present experimental setup for the specific investigation of PSMA radioligand therapy. It has to be noted, that only an ^18^F-labeled tracer has been used and no full dosimetry with a therapeutic tracer has been performed. Nevertheless, the current findings point to the inherent importance of a strictly theranostic approach for the investigation of PSMA radioligand therapy, since PSMA uptake does not appear to be present in all murine GBM, consistent with a highly heterogeneous PSMA expression in human GBM (38). While tumor-to-background contrast has been reported to be high in multiple studies (17–21) the absolute uptake values between patients vary considerably [e.g. TBRs ranging from 4.07 to 134.8 (18)]. The relationship between PSMA expression and tracer uptake remains unclear. An ongoing prospective clinical trial aims to contribute to the discussion by evaluating the correlation between PSMA expression and ^18^F-PSMA-1007 uptake in first-diagnosed GBM patients (NCT04588454) (39).

**Figure 6:**
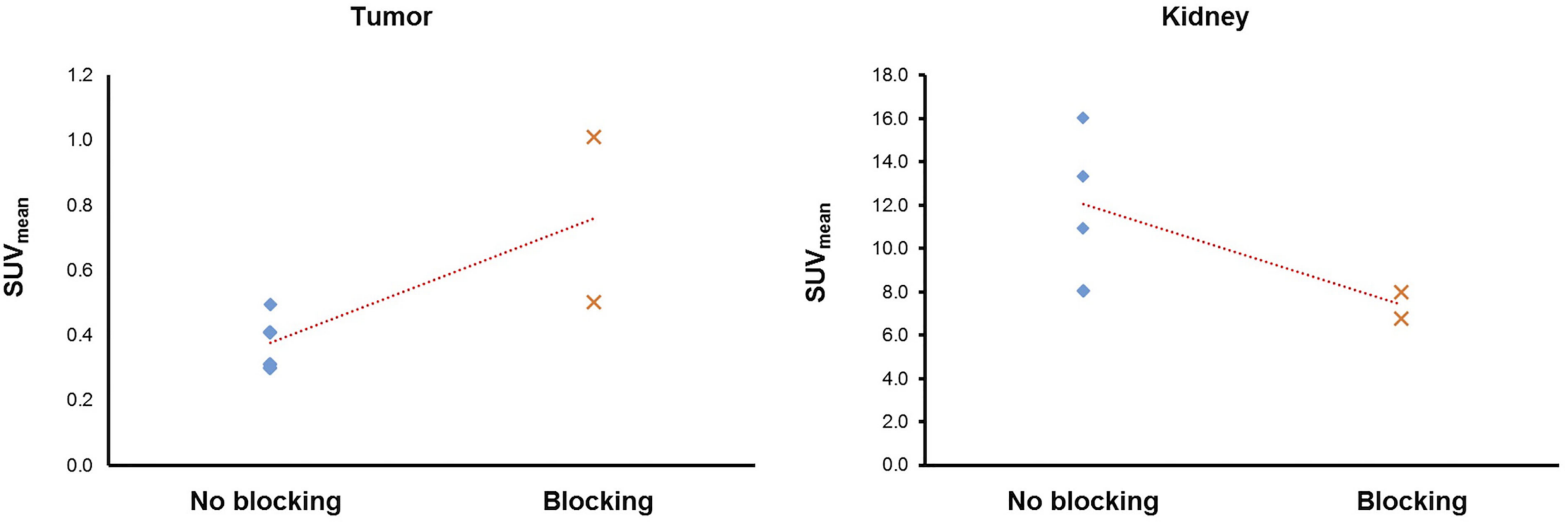
Tumor (left) and kidney (right) ^18^F-PSMA-1007 uptake in blocked and unblocked mice. Blocked mice received a bolus injection of cold PSMA reference standard prior to PET imaging with hot ^18^F-PSMA-1007. Surprisingly, the SUV_mean_ was lower in unblocked mice than in blocked mice. Kidney uptake decreased in blocked mice and confirms successful blocking (right).

Indeed, the present results lead us to hypothesize that the low uptake in the GL261 tumor model is most likely primarily due to a blood-brain-barrier (BBB) defect and not to specific binding to PSMA for the following reasons: First and foremost, the competitive binding assay indicated that the tumor signal in blocked mice is higher than the uptake in unblocked mice (**Figure 6**). We propose that this effect may occur due to higher tracer availability in the mouse blood because binding sites in mouse organs with high physiologic PSMA expression are blocked and therefore not available for peripheral tracer binding. While the results of this assay should not be overemphasized due to small number of cases which do not allow to statistically support our claim, the presented data indicates that blocking was not achieved in the tumor. Further, circumventricular organs which dispose of highly permeable capillaries showed high ^18^F-PSMA-1007 tracer accumulation in *ex vivo* ARG as well (**Figure 2F**), which is probably related to the absence of a BBB in this area. Thus, the increased PET signal in GBM might be analogously related to unspecific tracer accumulation in areas with disrupted BBB, although an additional specific uptake component cannot be excluded, as previously shown (32). Likewise, unspecific ^18^F-PSMA-1007 signal caused by a BBB defect has been reported before by Salas Fragomeni et al. (40) in a case of cerebral radionecrosis. Surprisingly, the *in vitro* ^18^F-PSMA-1007 ARG showed a decreased signal in the tumor area compared to the healthy brain tissue (**Figure 5**). This finding would also fit to unspecific ^18^F-PSMA-1007 signal within the tumor area in PET and in the *ex vivo* autoradiography. On the other hand, the negative *in vitro* ARG may simply be due to the fact that an *in vivo* process may be a key prerequisite for tumoral ^18^F-PSMA-1007 uptake, therefore leaving the significance of the *in vitro* ARG findings unclear with regard to the hypothesis. Also, it is unlikely that ^18^F-PSMA-1007 tracer exhibits impaired binding specificity to the murine PSMA protein in general, as we were able to block ^18^F-PSMA-1007 uptake in the kidney, which in the C57BL/6 is known to express the murine PSMA protein (41, 42).

In sum, the commonly used GL261 model, although resulting in high tumor-to-background contrast on PSMA PET imaging, harbors potential limitations with regard to the application of PSMA radioligand therapy. Presumably, a more detailed understanding of the pathophysiologic mechanisms for assumed specific and nonspecific PSMA uptake in GBM will be helpful when aiming for PSMA radioligand therapy. Although we were able to demonstrate that the GL261 model displays endothelial cells (see CD31 immunohistochemical staining in **Supplementary Material**) inclusion of additional tumor models with differential vascular properties as well as models which allow for a selective manipulation of the BBB may provide additional insights to this end. Here, incubation media-specific properties have to be acknowledged as well, as they may impact on the degree of infiltrative growth and BBB integrity and therefore contribute to mirror a more reliable *in vivo* biology of human glioblastomas as compared to the GL261 model used in the current study (43). For the evaluation of PSMA radioligand therapy in genreal, but especially in more infiltrative models, dual tracer studies including both PSMA ligands and amino tracers such as FET should be envisaged, in order to better take into account the non-contrast enhancing tumor volume (44). Further preclinical investigations into PSMA PET and radioligand therapy in GBM are warranted.

## Conclusion

^18^F-PSMA-1007 PET imaging of GBM results in high TBRs, as in the current study for the first time shown for the GL261 model, being one of the most frequently used GBM mouse models. However, the high contrast is predominantly due to low PSMA uptake in healthy brain tissue. Absolute tumoral PSMA uptake in PET instead remained low which hints to a limited eligibility of the GL261 model for an application of PSMA radioligand therapy. Therefore, further investigations into PSMA in GBM mouse models are warranted in order to establish a preclinical model for the evaluation of PSMA radioligand therapy.

## Data Availability Statement

The raw data supporting the conclusions of this article will be made available by the authors, without undue reservation.

## Ethics Statement

The animal study was reviewed and approved by Regierung von Oberbayern.

## Author Contributions

MAK and AH drafted the manuscript under the guidance of NLA and MB with further input from all co-authors. MO and KL prepared the cells and performed surgery. MAK, AH, DP, and LG performed PET imaging, cryosections and autoradiography. MAK, DP, and LG performed cell culture experiments. VCR performed H&E staining. RG, REK and EB performed immunohistochemical staining. MAK, MO, and KL performed CT imaging. MAK and AH performed image analysis and statistical analyses. KS, HI, JM, JN, SL, RG, REK, LMM, LB, LK, CB, and PB and increased the intellectual content. JM was not involved in performing the animal experiments. MB, KL, and NLA supervised the project. All authors contributed to the article and approved the submitted version.

## Funding

MK received a doctoral fellowship from Förderprogramm für Forschung und Lehre (FöFoLe). AH received a grant from Friedrich-Baur-Stiftung related to this project. NA thanks the Else Kröner Fresenius-Stiftung for the support of her research. This work included collaboration within the CRC 824 (research area B2 and Z2) of the German Research Foundation (DFG), project number 68647618, as well as the Research Group 2858, project number 421887978.

## Conflict of Interest

The authors declare that the research was conducted in the absence of any commercial or financial relationships that could be construed as a potential conflict of interest.

## Publisher’s Note

All claims expressed in this article are solely those of the authors and do not necessarily represent those of their affiliated organizations, or those of the publisher, the editors and the reviewers. Any product that may be evaluated in this article, or claim that may be made by its manufacturer, is not guaranteed or endorsed by the publisher.

## Abbreviations

ARG, Autoradiography; BG, Background; CLS, Cell Line Services; CPM, Counts per minute; CT, Computed tomography; DMEM/F-12, Dulbecco’s Modified Eagle Medium: Nutrient Mixture F-12; DMSO, Dimethyl sulfoxide; FCS, fetal calf serum; GBM, Glioblastoma multiforme; GT VOI, General tumor VOI; H&E, Haematoxylin and Eosin; HEPES, (4-(2-hydroxyethyl)-1-piperazineethanesulfonic acid; Iso_50_, 50% isocontour; PBS, Phosphate-buffered saline; PET, Positron emission tomography; PSMA, Prostate specific membrane antigen; ROI, Region-of-interest; RPMI, Roswell Park Memorial Institute; STR,Short tandem repeats; SUV, Standardized uptake value; TAC, Time-activity-curves; TBmR, Tumor-to-bone marrow ratio; TBR, Tumor-to-background ratio; TKR, Tumor-to-kidney ratio; TPR, Tumor-to-parotid gland ratio; TRIS, Tris(2-carboxyethyl)phosphin –hydrochlorid; TTP, Time-to-peak; VOI, Volume-of-interest.

## References

[1] Bradley CA. (68)Ga-PSMA-11 PET Enables Accurate Detection of Recurrent Disease. Nat Rev Clin Oncol (2019) 16(7):403. doi: 10.1038/s41585-019-0181-7 30952962

[2] Perera M Papa N Roberts M Williams M Udovicich C Vela I. Gallium-68 Prostate-Specific Membrane Antigen Positron Emission Tomography in Advanced Prostate Cancer-Updated Diagnostic Utility, Sensitivity, Specificity, and Distribution of Prostate-Specific Membrane Antigen-Avid Lesions: A Systematic Review and Meta-Analysis. Eur Urol (2020) 77(4):403–17. doi: 10.1016/j.eururo.2019.01.049 30773328

[3] Virgolini I Decristoforo C Haug A Fanti S Uprimny C. Current Status of Theranostics in Prostate Cancer. Eur J Nucl Med Mol Imaging (2018) 45(3):471–95. doi: 10.1007/s00259-017-3882-2 PMC5787224 29282518

[4] Bradley CA. [177lu]PSMA-617 Radionuclide Therapy Shows Promise. Nat Rev Urol (2018) 15(8):468. doi: 10.1038/s41585-018-0029-6 29789593

[5] Hofman MS Violet J Hicks RJ Ferdinandus J Thang SP Akhurst T. [(177)Lu]-PSMA-617 Radionuclide Treatment in Patients With Metastatic Castration-Resistant Prostate Cancer (LuPSMA Trial): A Single-Centre, Single-Arm, Phase 2 Study. Lancet Oncol (2018) 19(6):825–33. doi: 10.1016/S1470-2045(18)30198-0 29752180

[6] Parker C Nilsson S Heinrich D Helle SI O'Sullivan JM Fosså SD. Alpha Emitter Radium-223 and Survival in Metastatic Prostate Cancer. N Engl J Med (2013) 369(3):213–23. doi: 10.1056/NEJMoa1213755 23863050

[7] Siva S Callahan J Pryor D Martin J Lawrentschuk N Hofman MS. Utility of (68) Ga Prostate Specific Membrane Antigen - Positron Emission Tomography in Diagnosis and Response Assessment of Recurrent Renal Cell Carcinoma. J Med Imaging Radiat Oncol (2017) 61(3):372–8. doi: 10.1111/1754-9485.12590 28116853

[8] Volter F Mittlmeier L Gosewisch A Brosch-Lenz J Gildehaus FJ Zacherl MJ. Correlation of an Index-Lesion-Based SPECT Dosimetry Method With Mean Tumor Dose and Clinical Outcome After (177)Lu-PSMA-617 Radioligand Therapy. Diagnostics (Basel) (2021) 11(3):428. doi: 10.3390/diagnostics11030428 33802417 PMC7999994

[9] Nomura N Pastorino S Jiang P Lambert G Crawford JR Gymnopoulos M. Prostate Specific Membrane Antigen (PSMA) Expression in Primary Gliomas and Breast Cancer Brain Metastases. Cancer Cell Int (2014) 14(1):26. doi: 10.1186/1475-2867-14-26 24645697 PMC3994554

[10] Wernicke AG Edgar MA Lavi E Liu H Salerno P Bander NH. Prostate-Specific Membrane Antigen as a Potential Novel Vascular Target for Treatment of Glioblastoma Multiforme. Arch Pathol Lab Med (2011) 135(11):1486–9. doi: 10.5858/arpa.2010-0740-OA 22032578

[11] Matsuda M Ishikawa E Yamamoto T Hatano K Joraku A Iizumi Y. Potential Use of Prostate Specific Membrane Antigen (PSMA) for Detecting the Tumor Neovasculature of Brain Tumors by PET Imaging With (89)Zr-Df-IAB2M Anti-PSMA Minibody. J Neurooncol (2018) 138(3):581–9. doi: 10.1007/s11060-018-2825-5 29524126

[12] Chang SS Reuter VE Heston WD Bander NH Grauer LS Gaudin PB. Five Different Anti-Prostate-Specific Membrane Antigen (PSMA) Antibodies Confirm PSMA Expression in Tumor-Associated Neovasculature. Cancer Res (1999) 59(13):3192–8. 10397265

[13] Wen PY Weller M Lee EQ Alexander BM Barnholtz-Sloan JS Barthel FP. Glioblastoma in Adults: A Society for Neuro-Oncology (SNO) and European Society of Neuro-Oncology (EANO) Consensus Review on Current Management and Future Directions. Neuro Oncol (2020) 22(8):1073–113. doi: 10.1093/neuonc/noaa106 PMC7594557 32328653

[14] Tesileanu CMS Dirven L Wijnenga MMJ Koekkoek JAF Vincent A Dubbink HJ. Survival of Diffuse Astrocytic Glioma, IDH1/2 Wildtype, With Molecular Features of Glioblastoma, WHO Grade IV: A Confirmation of the cIMPACT-NOW Criteria. Neuro Oncol (2020) 22(4):515–23. doi: 10.1093/neuonc/noz200 PMC7158657 31637414

[15] Lu VM Goyal A Graffeo CS Perry A Burns TC Parney IF. Survival Benefit of Maximal Resection for Glioblastoma Reoperation in the Temozolomide Era: A Meta-Analysis. World Neurosurg (2019) 127:31–7. doi: 10.1016/j.wneu.2019.03.250 30947000

[16] Ostrom QT Patil N Cioffi G Waite K Kruchko C Barnholtz-Sloan JS. CBTRUS Statistical Report: Primary Brain and Other Central Nervous System Tumors Diagnosed in the United States in 2013-2017. Neuro Oncol (2020) 22(12 Suppl 2):iv1–iv96. doi: 10.1093/neuonc/noaa200 33123732 PMC7596247

[17] Bertagna F Albano D Cerudelli E Gazzilli M Giubbini R Treglia G. Potential of Radiolabelled PSMA PET/CT or PET/MRI Diagnostic Procedures in Gliomas/Glioblastomas. Curr Radiopharm (2019) 13(2):94–8. doi: 10.2174/1874471012666191017093721 PMC7527542 31625482

[18] Sasikumar A Kashyap R Joy A Charan Patro K Bhattacharya P Reddy Pilaka VK. Utility of 68Ga-PSMA-11 PET/CT in Imaging of Glioma-A Pilot Study. Clin Nucl Med (2018) 43(9):e304–9. doi: 10.1097/RLU.0000000000002175 29939953

[19] Unterrainer M Niyazi M Ruf V Bartenstein P Albert NL. The Endothelial Prostate-Specific Membrane Antigen Is Highly Expressed in Gliosarcoma and Visualized by [68Ga]-PSMA-11 PET: A Theranostic Outlook for Brain Tumor Patients? Neuro Oncol (2017) 19(12):1698–9. doi: 10.1093/neuonc/nox172 PMC5716188 29045711

[20] Marafi F Sasikumar A Fathallah W Esmail A. 18f-PSMA 1007 Brain PET/CT Imaging in Glioma Recurrence. Clin Nucl Med (2019) 45(1):e61–2. doi: 10.1097/RLU.0000000000002668 31162269

[21] Verma P Malhotra G Goel A Rakshit S Chandak A Chedda R. Differential Uptake of 68Ga-PSMA-HBED-CC (PSMA-11) in Low-Grade *Versus* High-Grade Gliomas in Treatment-Naive Patients. Clin Nucl Med (2019) 44(5):e318–22. doi: 10.1097/RLU.0000000000002520 30829867

[22] Stegen B Nieto A Albrecht V Maas J Orth M Neumaier K. Contrast-Enhanced, Conebeam CT-Based, Fractionated Radiotherapy and Follow-Up Monitoring of Orthotopic Mouse Glioblastoma: A Proof-of-Concept Study. Radiat Oncol (2020) 15(1):19. doi: 10.1186/s13014-020-1470-2 31969174 PMC6977274

[23] Cardinale J Schafer M Benesova M Bauder-Wust U Leotta K Eder M. Preclinical Evaluation of (18)F-PSMA-1007, A New Prostate-Specific Membrane Antigen Ligand for Prostate Cancer Imaging. J Nucl Med (2017) 58(3):425–31. doi: 10.2967/jnumed.116.181768 27789722

[24] Cardinale J Roscher M Schafer M Geerlings M Benesova M Bauder-Wust U. Development of PSMA-1007-Related Series of (18)F-Labeled Glu-Ureido-Type PSMA Inhibitors. J Med Chem (2020) 63(19):10897–907. doi: 10.1021/acs.jmedchem.9b01479 32852205

[25] Holzgreve A Brendel M Gu S Carlsen J Mille E Boning G. Monitoring of Tumor Growth With [(18)F]-FET PET in a Mouse Model of Glioblastoma: SUV Measurements and Volumetric Approaches. Front Neurosci (2016) 10:260. doi: 10.3389/fnins.2016.00260 27378835 PMC4906232

[26] Mhawech-Fauceglia P Zhang S Terracciano L Sauter G Chadhuri A Herrmann FR. Prostate-Specific Membrane Antigen (PSMA) Protein Expression in Normal and Neoplastic Tissues and Its Sensitivity and Specificity in Prostate Adenocarcinoma: An Immunohistochemical Study Using Mutiple Tumour Tissue Microarray Technique. Histopathology (2007) 50(4):472–83. doi: 10.1111/j.1365-2559.2007.02635.x 17448023

[27] Mahzouni P Shavakhi M. Prostate-Specific Membrane Antigen Expression in Neovasculature of Glioblastoma Multiforme. Adv BioMed Res (2019) 8:18. doi: 10.4103/abr.abr_209_18 30993088 PMC6425742

[28] Liu T Jabbes M Nedrow-Byers JR Wu LY Bryan JN Berkman CE. Detection of Prostate-Specific Membrane Antigen on HUVECs in Response to Breast Tumor-Conditioned Medium. Int J Oncol (2011) 38(5):1349–55. doi: 10.3892/ijo.2011.946 21331445

[29] Nguyen DP Xiong PL Liu H Pan S Leconet W Navarro V. Induction of PSMA and Internalization of an Anti-PSMA mAb in the Vascular Compartment. Mol Cancer Res (2016) 14(11):1045–53. doi: 10.1158/1541-7786.MCR-16-0193 27458033

[30] Sasikumar A Joy A Pillai MR Nanabala R Anees KM Jayaprakash PG. Diagnostic Value of 68Ga PSMA-11 PET/CT Imaging of Brain Tumors-Preliminary Analysis. Clin Nucl Med (2017) 42(1):e41–8. doi: 10.1097/RLU.0000000000001451 27846000

[31] Kunikowska J Kulinski R Muylle K Koziara H Krolicki L. 68Ga-Prostate-Specific Membrane Antigen-11 PET/CT: A New Imaging Option for Recurrent Glioblastoma Multiforme? Clin Nucl Med (2020) 45(1):11–8. doi: 10.1097/RLU.0000000000002806 31663868

[32] Oliveira D Stegmayr C Heinzel A Ermert J Neumaier B Shah NJ. High Uptake of 68Ga-PSMA and 18F-DCFPyL in the Peritumoral Area of Rat Gliomas Due to Activated Astrocytes. EJNMMI Res (2020) 10(1):55. doi: 10.1186/s13550-020-00642-0 32451793 PMC7378136

[33] Law I Albert NL Arbizu J Boellaard R Drzezga A Galldiks N. Joint EANM/EANO/RANO Practice Guidelines/SNMMI Procedure Standards for Imaging of Gliomas Using PET With Radiolabelled Amino Acids and [(18)F]FDG: Version 1.0. Eur J Nucl Med Mol Imaging (2019) 46(3):540–57. doi: 10.1007/s00259-018-4207-9 PMC6351513 30519867

[34] Vettermann F Suchorska B Unterrainer M Nelwan D Forbrig R Ruf V. Non-Invasive Prediction of IDH-Wildtype Genotype in Gliomas Using Dynamic (18)F-FET PET. Eur J Nucl Med Mol Imaging (2019) 46(12):2581–9. doi: 10.1007/s00259-019-04477-3 31410540

[35] Schottelius M Wurzer A Wissmiller K Beck R Koch M Gorpas D. Synthesis and Preclinical Characterization of the PSMA-Targeted Hybrid Tracer PSMA-I&F for Nuclear and Fluorescence Imaging of Prostate Cancer. J Nucl Med (2019) 60(1):71–8. doi: 10.2967/jnumed.118.212720 PMC6354225 30237214

[36] Kunikowska J Charzyńska I Kuliński R Pawlak D Maurin M Królicki L. Tumor Uptake in Glioblastoma Multiforme After IV Injection of [(177)Lu]Lu-PSMA-617. Eur J Nucl Med Mol Imaging (2020) 47(6):1605–6. doi: 10.1007/s00259-020-04715-z PMC7188710 32040612

[37] Weineisen M Schottelius M Simecek J Baum RP Yildiz A Beykan S. 68Ga- and 177Lu-Labeled PSMA I&T: Optimization of a PSMA-Targeted Theranostic Concept and First Proof-Of-Concept Human Studies. J Nucl Med (2015) 56(8):1169–76. doi: 10.2967/jnumed.115.158550 26089548

[38] Holzgreve A Biczok A Ruf VC Liesche-Starnecker F Steiger K Kirchner MA. PSMA Expression in Glioblastoma as a Basis for Theranostic Approaches: A Retrospective, Correlational Panel Study Including Immunohistochemistry, Clinical Parameters and PET Imaging. Front Oncol (2021) 11:646387. doi: 10.3389/fonc.2021.646387 33859946 PMC8042319

[39] 18F-PSMA PET/CT for Visualization of Glioblastoma Multiforme. Available at: https://ClinicalTrials.gov/show/NCT04588454.

[40] Salas Fragomeni RA Pienta KJ Pomper MG Gorin MA Rowe SP. Uptake of Prostate-Specific Membrane Antigen-Targeted 18f-DCFPyL in Cerebral Radionecrosis: Implications for Diagnostic Imaging of High-Grade Gliomas. Clin Nucl Med (2018) 43(11):e419–21. doi: 10.1097/RLU.0000000000002280 30247210

[41] Knedlik T Vorlova B Navratil V Tykvart J Sedlak F Vaculin S. Mouse Glutamate Carboxypeptidase II (GCPII) has a Similar Enzyme Activity and Inhibition Profile But a Different Tissue Distribution to Human GCPII. FEBS Open Bio (2017) 7(9):1362–78. doi: 10.1002/2211-5463.12276 PMC5586342 28904865

[42] Bacich DJ Pinto JT Tong WP Heston WD. Cloning, Expression, Genomic Localization, and Enzymatic Activities of the Mouse Homolog of Prostate-Specific Membrane Antigen/NAALADase/folate Hydrolase. Mamm Genome (2001) 12(2):117–23. doi: 10.1007/s003350010240 11210180

[43] Lee J Kotliarova S Kotliarov Y Li A Su Q Donin NM. Tumor Stem Cells Derived From Glioblastomas Cultured in bFGF and EGF More Closely Mirror the Phenotype and Genotype of Primary Tumors Than do Serum-Cultured Cell Lines. Cancer Cell (2006) 9(5):391–403. doi: 10.1016/j.ccr.2006.03.030 16697959

[44] Grossman SA Romo CG Rudek MA Supko J Fisher J Nabors LB. Baseline Requirements for Novel Agents Being Considered for Phase II/III Brain Cancer Efficacy Trials: Conclusions From the Adult Brain Tumor Consortium's First Workshop on CNS Drug Delivery. Neuro Oncol (2020) 22(10):1422–4. doi: 10.1093/neuonc/noaa142 PMC7566550 32506123

