# Supplementary material for: PSMA PET Imaging in Glioblastoma: A Preclinical Evaluation and Theranostic Outlook
Source: Front Oncol. 2021 Nov 17;11:774017. doi: 10.3389/fonc.2021.774017 (PMC8635528; doi:10.3389/fonc.2021.774017)
Supplement: Supplementary file 1 [file DataSheet_1.pdf]

Supplementary Material

Time-activity-curves (TACs) in tumor and dose-relevant organs

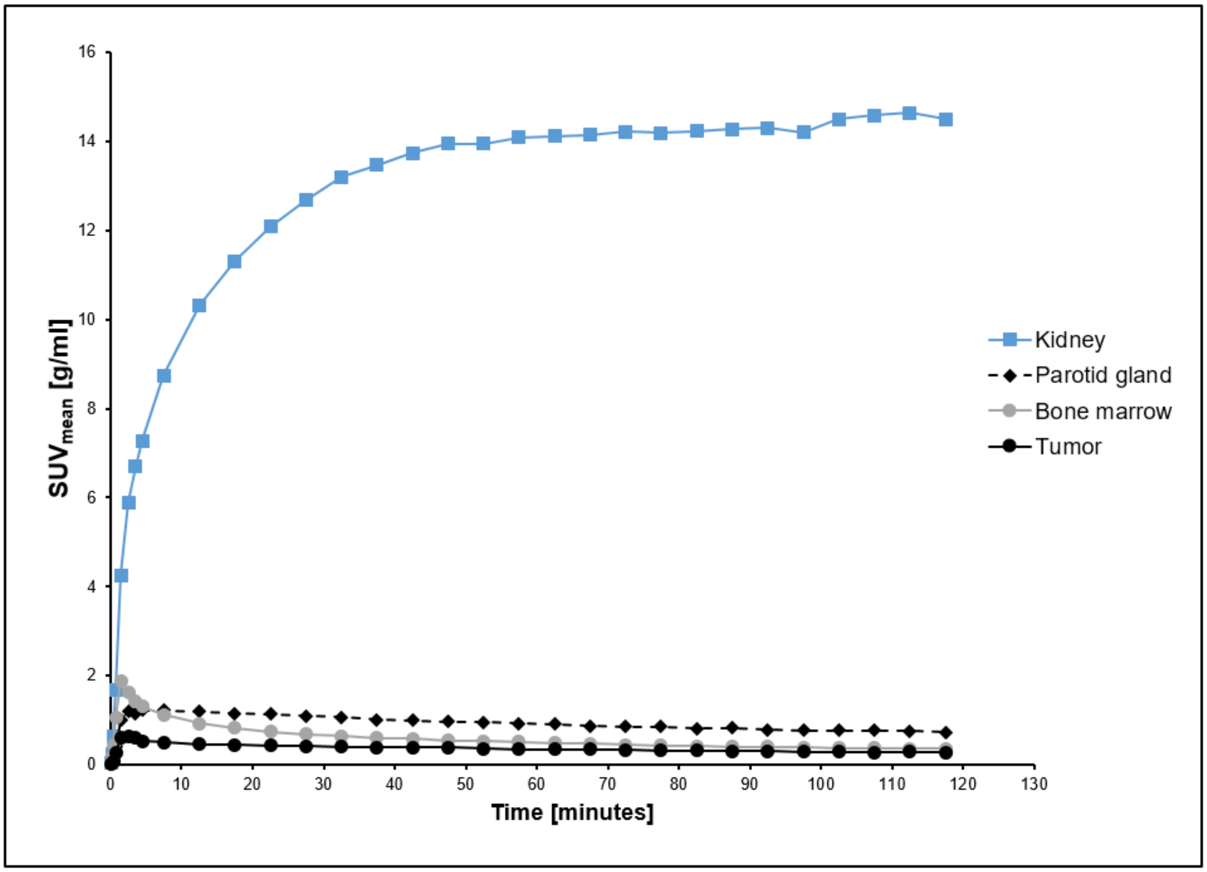

Kidney, parotid gland, bone marrow uptake compared to tumor uptake. TACs show average SUV_{mean} for n=4 GBM mice in the respective tissue. Average TTPs are 112.5 min, 7.5 min, 1.5 min 2.5 min for kidney, parotid gland bone marrow and tumor respectively.

CD31 immunohistochemical staining

Additional 40 µm formalin-fixed mouse brain cryosection specimens of a GL261 tumor bearing C57BL/6 mice were used for exemplary immunohistochemical CD31 endothelial staining. Immunohistochemical CD31 staining was performed as previously described (1). In brief, one mouse was euthanized on day 17 post inoculation and perfused with PBS and 4% paraformaldehyde, dehydrated and frozen at -20 °C in cryo-embedding solution (Tissue-Tec O.C.T.Sakura-Finetek). Overnight incubation at 4 °C with primary antibody (Rat anti-CD31, 550274 Becton Dickinson) was followed by 3 hour secondary antibody incubation (Alexa 647 donkey anti rat, Jackson Immuno Research, 712-605-153).

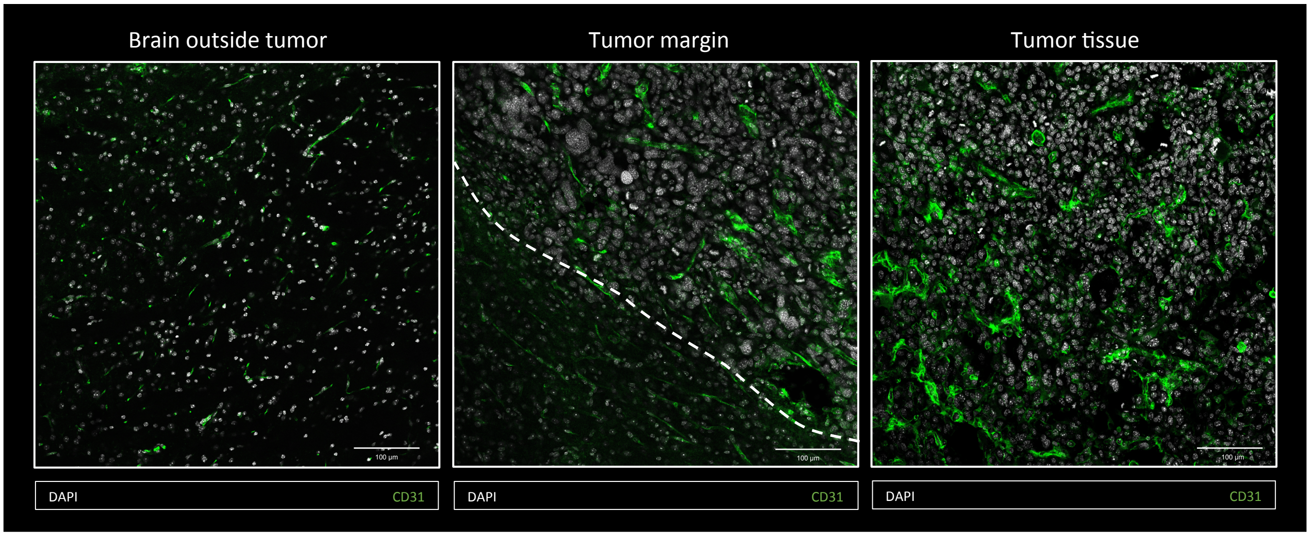

CD31 immunohistochemical staining. CD31 expression corresponding to endothelial cells can be found in GL261 tumor tissue. The tumor tissue displays a sharp demarcation to healthy brain tissue at the tumor margin. The tumor margin includes cells with modified morphology which might correspond to a reactive zone of astrocytic activation.

References

1. Kälin RE, Cai L, Li Y, Zhao D, Zhang H, Cheng J, et al. TAMEP are brain tumor parenchymal cells controlling neoplastic angiogenesis and progression. Cell systems. 2021;12(3):248-62.e7.
